# Genetic Diversity and Population Structure of Cucumber (*Cucumis sativus* L.)

**DOI:** 10.1371/journal.pone.0046919

**Published:** 2012-10-12

**Authors:** Jing Lv, Jianjian Qi, Qiuxiang Shi, Di Shen, Shengping Zhang, Guangjin Shao, Hang Li, Zhanyong Sun, Yiqun Weng, Yi Shang, Xingfang Gu, Xixiang Li, Xiaoguo Zhu, Jinzhe Zhang, Robbert van Treuren, Willem van Dooijeweert, Zhonghua Zhang, Sanwen Huang

**Affiliations:** 1 Institute of Vegetables and Flowers, Chinese Academy of Agricultural Sciences, Sino-Dutch Joint Lab of Horticultural Genomics, Opening Lab of Genetic Improvement of Agricultural Crops of Ministry of Agriculture, Beijing, China; 2 Middlebury College, Middlebury, Vermont, United States of America; 3 High School Affiliated to Renmin University of China, Beijing, China; 4 East-West Seed International Ltd., Nanning, Guangxi, China; 5 United States Department of Agriculture (USDA), ARS, Vegetable Crops Research Unit, Department of Horticulture, University of Wisconsin, Madison, Wisconsin, United States of America; 6 Centre for Genetic Resources, The Netherlands, Wageningen University and Research Centre, Wageningen, The Netherlands; Nanjing Agricultural University, China

## Abstract

Knowing the extent and structure of genetic variation in germplasm collections is essential for the conservation and utilization of biodiversity in cultivated plants. Cucumber is the fourth most important vegetable crop worldwide and is a model system for other Cucurbitaceae, a family that also includes melon, watermelon, pumpkin and squash. Previous isozyme studies revealed a low genetic diversity in cucumber, but detailed insights into the crop's genetic structure and diversity are largely missing. We have fingerprinted 3,342 accessions from the Chinese, Dutch and U.S. cucumber collections with 23 highly polymorphic Simple Sequence Repeat (SSR) markers evenly distributed in the genome. The data reveal three distinct populations, largely corresponding to three geographic regions. Population 1 corresponds to germplasm from China, except for the unique semi-wild landraces found in Xishuangbanna in Southwest China and East Asia; population 2 to Europe, America, and Central and West Asia; and population 3 to India and Xishuangbanna. Admixtures were also detected, reflecting hybridization and migration events between the populations. The genetic background of the Indian germplasm is heterogeneous, indicating that the Indian cucumbers maintain a large proportion of the genetic diversity and that only a small fraction was introduced to other parts of the world. Subsequently, we defined a core collection consisting of 115 accessions and capturing over 77% of the SSR alleles. Insight into the genetic structure of cucumber will help developing appropriate conservation strategies and provides a basis for population-level genome sequencing in cucumber.

## Introduction

Commonly known as cucurbits or gourds, the botanical family Cucurbitaceae includes a number of cultivated species of global or local economical importance [1]. Cucumber (*Cucumis sativus* L.) is the fourth most important vegetable worldwide [2]. As the first in cucurbits, the cucumber genome sequence elucidated chromosomal evolution in the genus *Cucumis* and afforded novel insights into several important biological processes such as biosynthesis of cucurbitacin and “fresh green” odor. Cucumber is being developed as a new model species in plant biology due to its small number of genes, rich diversity of sex expression, suitability for vascular biology studies, short life cycle (three months from seed to seed), and accumulating resources in genetics [3], [4] and genomics [5], [6].

The rapid advance of Next Generation Sequencing (NGS) technologies makes it affordable to re-sequence multiple genotypes of a given species to generate a haplotype map that displays the genome-wide patterns of genetic variation at a single base resolution [7], [8], [9]. However, such population sequencing requires a well-designed sampling of the species' genetic diversity. To date, little is known about the genetic diversity and structure in cucumber.

Cucumber is indigenous to India [10] and likely originated from the foothills of the Himalayan Mountain, where its only two botanical varieties both were discovered, namely the domesticated cucumber *C.s.* var. *sativus* and the wild cucumber *C.s.* var. *hardwickii* (Royle) Alef. The cultivation of cucumber seems to have spread rapidly from India to Western Asia, and then to Southern Europe. It has been reported that cucumber was introduced into China through the Silk Route by the diplomat Zhang Qian (164 B.C.–114 B.C.) during the Han Dynasty and subsequently spread to East Asia [11]. A unique semi-wild landrace, named Xishuangbanna Gourd, was discovered in the Prefecture Xishuangbanna of the Province Yunnan in Southwest China. To date, its relationship to other phylogeographic populations is unknown.

Genetic diversity in the U.S. cucumber collection (∼1,000 Plant Introductions, or PIs) maintained by the National Plant Germplasm System (NPGS) was investigated in a series of studies by analysis of variation at 21 isozyme loci [12], [13], [14], [15]. Overall, these studies revealed low diversity (∼2.2 alleles per polymorphic locus) in domesticated cucumber. Possibly due to the lack of polymorphism, the isozyme data did not allow consistent clustering of the PIs. Consequently, the genetic structure of domesticated cucumber remained largely unknown.

In this study we assembled a mega-collection consisting of all available cucumber accessions from the national germplasm centres of China, the Netherlands and U.S. We fingerprinted a total of 3,342 accessions with 23 highly polymorphic Simple Sequence Repeat (SSR) markers. The SSR data were used to assess overall genetic diversity and to reveal a clear population structure in cucumber. In addition, the data allowed the construction of a core set of 115 accessions capturing over 77% of the total allelic diversity observed.

## Materials and Methods

### Materials

A mega-collection of 3,342 cucumber accessions was used in this study. A total of 1,692 accessions were collected from the China National Vegetable Germplasm Bank located at the Institute of Vegetables and Flowers at the Chinese Academy of Agricultural Sciences (IVF-CAAS), 883 accessions from the U.S. National Plant Germplasm System (NPGS, http://www.ars-grin.gov/npgs/), 759 accessions from the Centre for Genetic Resources, the Netherlands (CGN, http://www.cgn.wur.nl), 2 accessions from the IPK (http://www.ipk-gatersleben.de/), and 6 accessions from the East-West Seed International Ltd. The mega-collection included 235 accessions originating from India, 1,361 from China (excluding Xishuangbanna), 208 from Xishuangbanna, 209 from Southeast Asia, 141 from Western Asia, 802 from Europe, 176 from the Americas, 50 from Africa and 8 from Oceania. Additionally, 152 accessions of unknown origin were included in the study. Passport information about the mega-collection is listed in Table S1.

### SSR analyses

#### DNA extraction

For most of the study accessions multiple self-crossing was performed during gene bank curation, and previous studies on cucumber germplasm have revealed limited intra-accession variation. To increase the efficiency in analysing the large number of accessions, leaves from five randomly chosen plants per accession were bulked into a single sample. Genomic DNA was isolated from about one gram of leaf tissue from each bulk using a CTAB-based method [16].

#### SSR markers

**Figure 1:**
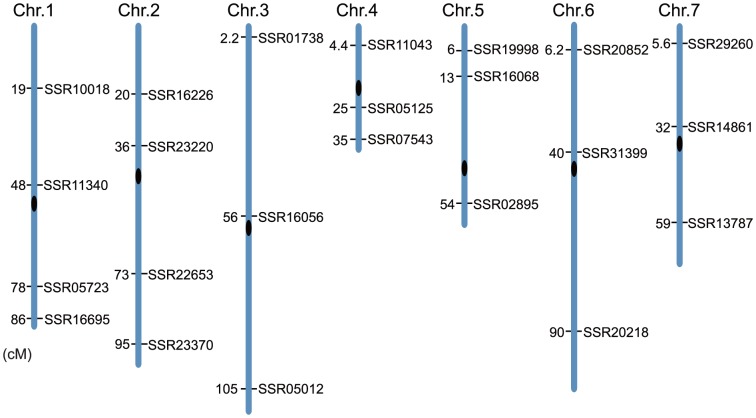
Position of the 23 SSR loci on the cucumber genetic map [18].

In previous studies [3], [4], we determined the polymorphism level of 995 SSR markers using a selected panel of 11 cucumber genotypes that represent six market types worldwide: ‘Chinese Long’, Southern China type, Xishuangbanna type, European greenhouse type, American slicing type, and Japanese type. For the present study, we selected 23 highly polymorphic SSR markers that are evenly distributed across the cucumber genetic map (Table 1). Each selected SSR marker is able to produce a clear, stable, and single polymerase chain reaction (PCR) product. On each chromosome arm, there is at least one SSR (Figure 1). The genetic distance between two neighbouring markers ranges from7.1 cM to 53.7 cM with an average of 29.4 cM.

**Table 1:** Characteristics of the 23 investigated SSR markers and the diversity detected in 3,342 cucumber accessions.

	Locus	cM	Motif	Primer sequence (5′to 3′)	Na	Ne	Ho	He	I	PIC
Chr1	SSR10018	19	(AAG)19	F:GGGTCTAATATTTGGGGATGG	16	3	0.29	0.60	1.3	0.65
				R:GGTTGTTCTTGTGGAATGTG						
	SSR11340	48	(AG)18	F:TTGTTTTTGTTGGGCACTCA	14	2	0.10	0.54	1	0.76
				R:GTGCATCACTCACCCCTTC						
	SSR05723	78	(AT)17	F:GGGTGTAATTGGCTTTTCTG	10	2	0.12	0.51	1.1	0.38
				R:GGTTCTAATCCAACGAGTGC						
	SSR16695	86	(CAT)14	F:GGACTAGAAACACAATCCCACG	7	2	0.13	0.52	0.8	0.39
				R:GTTTGGTTTGCTTCAAGTAGGTTC						
Chr2	SSR16226	20	(TCTT)7	F:GCGTTAAAATTCCCAACGG	8	3	0.23	0.67	1.2	0.62
				R:GGAGAGAAATTGGAATTCGGCAG						
	SSR23220	36	(AGAAGG)13	F:GGGATGGGATCTGGGTTTG	10	2	0.07	0.48	0.9	0.5
				R:GTGTGGAAATATGTGGAGGGAG						
	SSR22653	73	(AG)20	F:TGAATTTCTTTGGTGGATTCAA	14	3	0.23	0.61	1.2	0.55
				R:GGGAGAAGAAGGGGAGATTG						
	SSR23370	95	(TA)17	F:GATTATGAGGATGAACCACACC	13	3	0.31	0.70	1.5	0.76
				R:GCCAACAACTCTCTCTTATCGAAC						
Chr3	SSR01738	2.2	(GAA)9	F:GCGTAGGGAAAGTAAATCAAATAGG	13	2	0.15	0.50	0.9	0.42
				R:GGCATAAGAAATGATACGAACC						
	SSR16056	56	(CACCCT)6	F:GGGTTTGATAGTGGAGATTATTCA	15	4	0.29	0.74	1.6	0.68
				R:GGTCTTTTCCACTCAATCCATT						
	SSR05012	105	(TCT)8	F:TTTAATGGCGTCGAAATGGT	6	2	0.36	0.51	0.7	0.34
				R:GTTCCATTAACGAGCTTCCC						
Chr4	SSR11043	4.4	(GAA)16	F:TACACCTCTGCGAAGCACC	15	3	0.33	0.66	1.5	0.64
				R:GTTTCGCACTCACTCTTTACCG						
	SSR05125	25	(CACCCT)6	F:GCACATTCAAATTTACTTGGGAG	14	2	0.12	0.58	1.1	0.68
				R:GCTTTAAGTTTGATGGTAGGGTAG						
	SSR07543	35	(GA)13	F:GGTTTGGCTTTCCTTTCACTC	9	3	0.16	0.63	1.1	0.63
				R:GGTTCCCCAAATCAAACTCAC						
Chr5	SSR19998	6	(AATC)12	F:CTTTGCCAAGCATCTCACC	10	1	0.21	0.30	0.7	0.3
				R:GTTTGCGTCTGCGGTTCTG						
	SSR16068	13	(AG)12(AAAGAG)5	F:GTGCAAAACGGAGTGAGGTG	12	2	0.14	0.46	0.9	0.57
				R:GTTTGGGTTTGGTTCTGATG						
	SSR02895	54	(AT)19	F:GTGAAGAAATGAGTTGGCAAGTC	23	4	0.30	0.73	1.7	0.88
				R:GGAGGGAATGTTGGATCAGC						
Chr6	SSR20852	6.2	(TA)30	F:GGTTTCCATTGAACTCGTAGC	24	3	0.13	0.68	1.7	0.74
				R:GGCTGTCCATTTTGTAGAACC						
	SSR31399	40	(AT)18	F:AGCTCCGAGGATACCCATCT	10	3	0.18	0.61	1.1	0.67
				R:AGAAGAACACCTGGAACAGACA						
	SSR20218	90	(AGA)28	F:TCGCCCACGTCCTCTATATC	8	2	0.25	0.54	0.9	0.6
				R:GCTAATGAAGGGGGAGGAGA						
Chr7	SSR29620	5.6	(GAGATG)8	F:TGCTTGGAAGTTTGTCCTGTC	11	2	0.19	0.49	1	0.64
				R:GGTTTATTGGATGATGGGTC						
	SSR14861	32	(ATAC)19	F:CGGTAGTCTACTTGGTTGAAATG	16	3	0.15	0.59	1.3	0.7
				R:GTAAATAGGACGAAGGAAAACCAC						
	SSR13787	59	(AT)18	F:GCAACTCCAACCAATCCCTC	25	3	0.35	0.66	1.7	0.76
				R:GGCAGCTAAATTCAACTCACC						

Na: the observed number of alleles; Ne: the effective number of alleles; H_o_: observed heterozygosity; H_e_: expected heterozygosity; I: Shannon's information index; PIC: polymorphism information content.

#### PCR amplification

The 23 SSR markers were analyzed using four sets of multiplex PCR reactions. Each multiplex was carefully assembled according to the compatibility of the SSRs during PCR and the molecular size of their amplicons. The forward primer of the SSR markers was labelled with one of the four fluorescent dyes, carboxy fluorescein (FAM), carboxytetramethylrhodamine (TAMRA), hexachloro-6-carboxyfluorescein (HEX) and ROX (carboxy-X-rhodamine). Multiplex PCRs were performed in a 11 µl volume containing approximately 20–50 ng template DNA, 0.4 pmol of each primer, and 5 µl Multiplex PCR Master Mix (QIAGEN Multiplex PCR Kit, Qiagen). Reactions were performed in an ABI 9700 thermocycler with an initial denaturation step of 15 min at 95°C, followed by 35 cycles of 95°C for 30 s, 57°C for 90 s and 72°C for 70 s, and a final extension at 60°C for 30 min. For PCR fragment size determinations, 0.25 µl of an internal size standard (Liz-500, LIZ) was mixed with 1 µl of diluted PCR product (1/100) and 9 µl formamide. The mixture was heated at 94°C for 3 minutes and then cooled within icy water. Electrophoresis was carried out on an ABI 3130×l genetic analyzer (Applied Biosystems). Analyses were performed using the Genemapper 4.0 software.

### Data analysis

#### Genetic structure

The model-based program STRUCTURE [17] was used to infer population structure and to identify hybrid forms. In order to identify the number of populations (K) capturing the major structure in the data, we used a burn-in period of 10,000 Markov Chain Monte Carlo iterations and 100,000 run length, an admixture model following Hardy-Weinberg equilibrium and correlated allele frequencies as well as independent loci for each run. Three independent runs were performed for each simulated value of K, ranging from 1 to 20. Subsequently, an Pr(X|K) index with respect to each K was used to calculate ΔK using the formula described by Evanno et al. [18]. The optimal K depends on the first peak of ÄΔK = |L″(K)|/s[Pr(x|k)], where (|L″(K)| denotes the absolute value of the second order rate of change of Pr(X|K), and s[Pr(x|k)] the standard deviation of the Pr(X|K)).

#### Genetic diversity assessment

For the entire collection as well as the subpopulations, allele richness, gene diversity and the number of unique alleles were calculated with PowerMarker version 3.25 [19], using genetic distances calculated by the CS Chord 1967 method [20]. Tree topologies were constructed based on the neighbour-joining method with MEGA [21]. For each subpopulation inferred from STRUCTURE, the observed and effective number of alleles, the observed and expected heterozygosity, as well as Shannon's information index were calculated by POPGENE. As a measure of genetic differentiation between pairs of sub-populations, we computed *F_ST_* statistics with the AMOVA means in Arlequin 3.11 [22]. Principal Component Analysis (PCA) in NTSYSpc2.11 was used to analyse genetic relationships among accessions and to determine the optimal number of clusters in the study.

#### Core collection sampling

For sampling core collections, we used the Maximization (M) algorithm implemented in MSTRAT software version 4.1 [23]. For evaluation of core collection's minimal size and for individual sampling of the collections, 20 replicates of 20 iterations for each replicate were performed.

## Results

### Molecular fingerprinting of the mega-collection

We fingerprinted 3,342 cucumber accessions using 23 SSR markers. In total, 94.9% of PCR reactions yielded clear peaks in electrophoresis and generated 72,960 data points. The remaining 5.1% resulted in multiple bands with low quality and consequently were treated as missing values in subsequent analyses. Out of the 72,960 data points, 61,976 (84.9%) corresponded to single peaks that were regarded as homozygous for the given SSR locus, while 8,929 (12.2%) corresponded to double peaks that were regarded as heterozygous. Only 2,055 (2.8%) of the data points corresponded to more than two clear peaks, of which only the two highest peaks were recorded that were regarded as heterozygous. This simplified scoring may cause under-estimation of rare alleles.

The 23 SSR markers generated a total of 316 alleles in the mega-collection (Table 1). Out of the 316 SSR alleles, 64 displayed a frequency of more than 5% in the total sample and hence were classified as ‘common’ alleles, while another 64 displayed frequencies between 1% and 5% that were denoted as ‘less common’ alleles. Out of the 188 remaining alleles, 121 were denoted as ‘rare alleles’ with frequencies between 0.1% and 1% and 67 as ‘very rare’ alleles with frequencies smaller than 0.1%. That 59% of the observed alleles showed a frequency of less than 1% in the mega-collection suggested that this collection represents the bio-diversity of cucumber broadly.

### Genetic structure in cucumber

**Figure 2:**
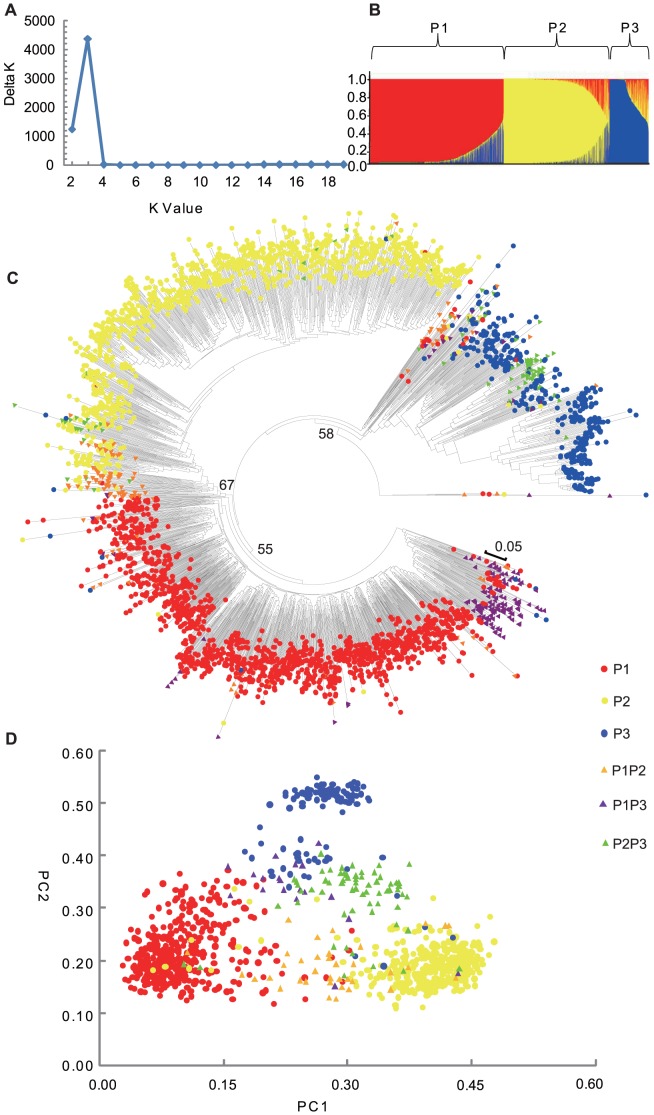
Model-based populations in the cucumber mega-population. (A) Delta K values for different numbers of populations assumed (K) in the STRUCURE analysis. (B) Classification of 3,342 accessions into three populations using STRUCTURE 2.3.1. The distribution of the accessions to different populations is indicated by the color code (P1: red, P2: yellow, P3: blue). Numbers on the y-axis show the subgroup membership, and the x-axis shows the different accession. (C) Unrooted Neighbor-Joining (NJ) tree of the 3,342 accessions. Bootstrap values are indicated at the major branches. (D) Principal Component Analysis (PCA) of the 1,026 accessions without missing SSR fingerprinting data. Color codes for each population and admixture are consistent in Figure 2C, 2D, Figure 3 and Figure 4C.

The population structure of the cucumber mega-collection was firstly inferred using STRUCTURE 2.3.1 [18]. The Evanno et al. (2005) correction showing only peak of ΔK, for K = 3, suggested the presence of three main populations in cucumbers (Figure 2A). The classification of accessions into populations by the model-based method was shown in Figure 2B and Table S1. In total 2,932 accessions (87.7%) were assigned to one of the three populations, where more than 70% of their inferred ancestry was derived from one of the model-based populations. Population 1, 2, and 3 (P1, P2, and P3) consisted of 1399, 1129, and 404 accessions, respectively. The remaining 410 accessions (12.3%) were categorized as having admixed ancestry, including 152 admixtures between P1 and P2 (P1P2), 163 between P1 and P3 (P1P3), and 95 between P2 and P3 (P2P3).

The unrooted neighbour-joining tree (Figure 2C) is consistent with the aforementioned model-based population structure. Accessions of the three populations are largely, but not completely, separated. Admixtures are mostly located in between two populations.

We also performed Principal Component Analysis (PCA) on 1,026 accessions without any missing SSR data (Figure 2D). This analysis largely supported the separation of the accessions into three populations. P3 can be divided into two subgroups, one consisting mainly of accessions from India and the other with accessions from Xishuangbanna. Most admixtures appear in between the two populations.

In summary, the model-based ancestry analysis, the phylogenetic tree and the PCA strongly supported that cucumber has three well-differentiated genetic populations and admixtures.

### Geographical distribution of cucumber populations

**Figure 3:**
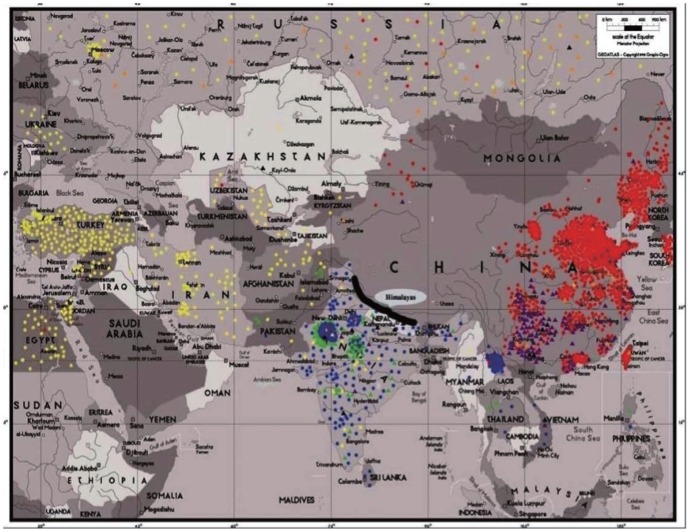
Geographical distribution of the investigated cucumber accessions. Color codes denote the populations shown in Figure 2.

The classification of populations appeared highly correlated with the geographical distribution of the cucumber accessions (Table 2, Figure 3). P1 includes cucumber germplasm from North and East China, Japan and South Korea, P2 includes accessions collected from Central and West Asia, Europe, America and Africa, while P3 comprises 404 accessions mainly from India and Xishuangbanna. Xishuangbanna is geographically close to the Indo-Gangetic plain where cucumber originated. Also the distribution of the admixtures appeared related to geography. About a quarter of the P1P2 admixtures (36 of 152) originated from East Europe including the Russian Federation and the Former Soviet Union. The P1P3 admixtures are mainly from Southwest China and India, and the P2P3 admixtures mainly from India. The admixtures are most likely due to natural or artificial hybridization.

**Table 2:** Geographic origin of the accessions assigned by the software STRUCURE to populations (P1–P3) and admixtures (P1P2–P2P3).

Region	P1	P2	P3	P1P2	P1P3	P2P3	Total
East Asia	1299	7	4	47	143	3	1503
Southeast Asia	0	8	41	2	5	11	67
Oceania	1	4	1	0	1	1	8
Africa	1	38	4	2	1	4	50
America	8	144	0	22	0	2	176
Europe	15	716	3	64	0	4	802
Central and West Asia	1	136	0	3	0	1	141
Xishuangbanna	1	0	205	0	2	0	208
India	1	16	134	7	11	66	235
Others	72	59	13	5	0	3	152
Total	1399	1129	404	152	163	95	3342

In PCA analysis (Figure 2D), P3 can be divided into two subgroups, one with accessions from India mainly and the other with accessions from Xishuangbanna. With more markers, the subgroups might be differentiated into two separate populations.

### Genetic diversity assessment

AMOVA analysis of the populations P1, P2 and P3 indicated that 68.1% of the variation was due to differences within populations, while 31.9% was due to differences among populations (Table 3). Pairwise estimates of *F_ST_* using AMOVA indicated a high degree of differentiation between the three model-based populations with values ranging from 0.30 to 0.33 (between P1 and P2: 0.33, P1 and P3: 0.30; P2 and P3: 0.31).

**Table 3:** Analysis of molecular variance (AMOVA) among populations and within populations.

Source of variation	d.f.	Sum of squares	Variance components	Percentage of variation
Among populations	2	9543.5	2.7	31.9
Within populations	5869	33551.8	5.7	68.1
Total	5871	43095.3	8.4	100

Genetic parameters for all accessions and the three model based populations are given in Table 4. For all accessions genotyped, the average number of alleles per locus was 13.70, ranging from 6 (SSR05012) to 25 (SSR13787) (Table 1). The Expected number of alleles was much smaller than that of observed, indicating the existence of few high frequency alleles in cucumber genome. The overall genetic diversity of cucumber was similar to tomato (0.2–0.58) [24] and rice (0.16–0.79) [25], but lower than maize (0.54–0.82) [26].

**Table 4:** Summary of SSR diversity parameters of the model-based populations and admixtures.

	P1	P2	P3	P1P2	P1P3	P2P3	All
Na	10.3	8.65	10.1	6.78	8.09	6.83	13.70
Ne	2.25	1.79	2.60	2.32	2.37	2.39	2.52
Ho	0.26	0.15	0.14	0.23	0.34	0.19	0.21
He	0.43	0.37	0.52	0.53	0.54	0.54	0.58
I	0.90	0.75	1.12	1.01	1.06	1.07	1.18
No. of unique alleles	17	3	40	0	5	2	

Na: the observed number of alleles; Ne: the effective number of alleles; H_o_: observed heterozygosity; H_e_: expected heterozygosity; I: Shannon's information index.

Among the three model based populations, P3 possessed the highest diversity (He = 0.52), though there was no significant differences in Na and Ne. Even with the least number of accessions involved, P3 displayed the largest number of population specific alleles (n = 40). This supports the notion that cucumber was originated from India [10].

For all populations, the observed heterozygosity was lower than expected heterozygosity, which may be caused by inbreeding during seed propagation by germplasm banks.

### Core Collection

**Figure 4:**
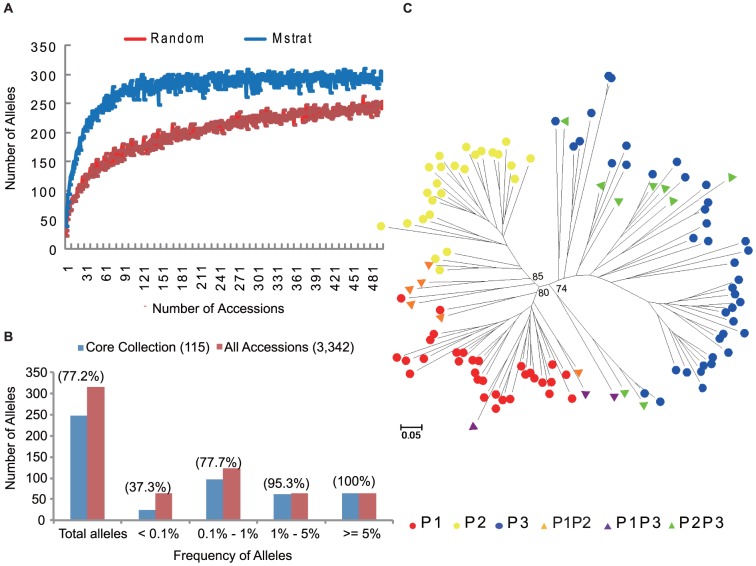
Development and evaluation of a core collection. (A) The minimized number of accessions for a core collection capturing the majority of alleles by random sampling and the Mstrat strategy. (B) Number of alleles observed in the total sample and those captured in the core collection, also presented separately for alleles occurring in different frequency. (C) NJ tree of the 115 core accessions. Values at the branches denote bootstrap values.

The Maximization or M strategy algorithm was used to select the core set of the mega-population. The M curve reached the plateau when approximately 115 accessions were selected (Figure 4A). Taking geographic distribution, existing phenotype data, and population structure into account, we selected 115 accessions to define the core collection (Table S2). The core collection captures 77.2% of the alleles discovered in the mega-collection, including all the 64 common alleles, 61 of the 64 less common alleles, 94 of the 121 rare alleles and 25 of the 67 very rare alleles (Figure 4B). The core collection has higher effective number of alleles, higher expected heterozygosity, and higher gene diversity than the entire collection (Table 5). In general, the topology of the unrooted neighbour-joining tree of the core collection (Figure 4C) resembles the tree of the mega-population, while better separating the populations. The P2P3 admixtures are clustered with Indian P3 accessions, indicating their evolutionary relationship.

**Table 5:** Genetic diversity parameters of the entire sample and those of the core collection based on the 23 investigated SSR loci.

	Na	Ne	I	He
All accessions	13.70	2.52	1.18	0.58
Core collection	10.65	3.24	1.44	0.66
Proportion of core collection to all	0.78	1.28	1.22	1.14

Na: the observed number of alleles; Ne: the effective number of alleles; H_o_: observed heterozygosity; H_e_: expected heterozygosity; I: Shannon's information index.

## Discussion

In this study, we carefully selected 23 highly polymorphic SSR markers and used them to fingerprint a large cucumber collection. The dataset allowed us to visualize the population structure of cucumber and to assemble a core collection of 115 accessions that cover over 77% of the observed SSR alleles.

Out of the 316 SSR alleles scored, 59% and 80% are present in less than 1% and 5% of the mega-collection, respectively. As the level of gene flow is negatively related to the number of rare alleles [27], the abundance of rare alleles in the mega-collection indicates that there has been limited genetic exchange among sub-populations in cucumber.

The results also supports that independent migrations of cucumber have occurred from India to other parts of the world. The migration route to Europe and then to the Americas and Africa are clearly through Central and West Asian countries. Cucumber accessions from China and other East Asian countries are genetically differentiated from those in Central and West Asia. It is well documented in Chinese history that cucumber was introduced from there through the Silk Route by the diplomat Zhang Qian (164 B.C.–114 B.C.) [11]. In addition, cucumber cultivation in heated houses during the winter season was recorded in poems from the Tang Dynasty (618–907). These records support that the crop has been intensively cultivated and therefore selected by Chinese farmers for more than one thousand years, which likely caused the genetic differentiation of Chinese (East Asian) cucumbers related to other populations.

Cucumber plants in India and Xishuangbanna bear short, ovary to round fruits with sparse spines (Figure S1). The occurrence of black spines, an unfavourable market trait, within Indian resources, is more frequent than the other two populations. Cucumbers should have undergone differential human selection when introduced to other parts of the world. For instance, in Europe and America, short-fruited gherkins and long fruited Dutch cucumbers were both developed to satisfy different market need (Figure S1). To enhance total yield, gynoecious varieties were widely adopted by western farmers as they could bear more fruits [28]. But in East Asia where cucumber is often served for direct consumption, characteristics such as proper appearance, crispness and “fresh green” flavour outweigh other traits (Figure S1). As a result, the ‘Chinese Long’ type had been developed to meet with that specific demand.

The Xishuangbanna cucumbers are affiliated to the Indian population. As semi-wild landraces, they apparently lost the unfavourable fruit bitterness but still possess the dependence of short daylight for flowering and fruit setting. The latter trait was discarded in most cucumber cultivars. Accessions from Xishuangbanna are genetically close to each other, suggesting that a single dispersal occurred from India. It is worth mentioning that among the African accessions, six lines are present from Zambia and Zimbabwe classified as P3 and admixture P2P3 (CG7743–CG7745, CG7747–CG7749, Table S1). The accessions were initially misclassified as melon and likely represent another dispersal from India.

The study also has potential implications for cucumber breeding. Currently, most crosses have been made within populations and less frequently among populations. The large differentiation among populations indicates that each population may possess its own alleles and haplotypes. Therefore, crosses between populations will broaden the genetic diversity within current breeding programs and may result in new types of heterosis. This study provides a sound basis for further characterization of the biodiversity of this important vegetable. For example, we are currently re-sequencing the core collection, enabling in-depth discovery of the genetic variation in cucumber.

## Supporting Information

Table S1 **Detail information of cucumber resources.**
(XLSX)

Table S2 **Cucumber core collection.**
(XLSX)

Figure S1 **Pictures of typical cucumber accessions from the three model based populations.** CG1149, “Chinese Long”; CG4357, Southern China type; CG5786, Dutch greenhouse; CG6600, Pickling; CG9191, Xishuangbanna; CG8039, Indian
(PDF)
