# Supplementary material for: Genetic Diversity and Population Structure of Cucumber (Cucumis sativus L.)
Source: PLoS One. 2012 Oct 12;7(10):e46919. doi: 10.1371/journal.pone.0046919 (PMC3470563; doi:10.1371/journal.pone.0046919)
Supplement: Figure S1 — Pictures of typical cucumber accessions from the three model based populations. CG1149, “Chinese Long”; CG4357, Southern China type; CG5786, Dutch greenhouse; CG6600, Pickling; CG9191, Xishuangbanna; CG8039, Indian (PDF) [file pone.0046919.s003.pdf]

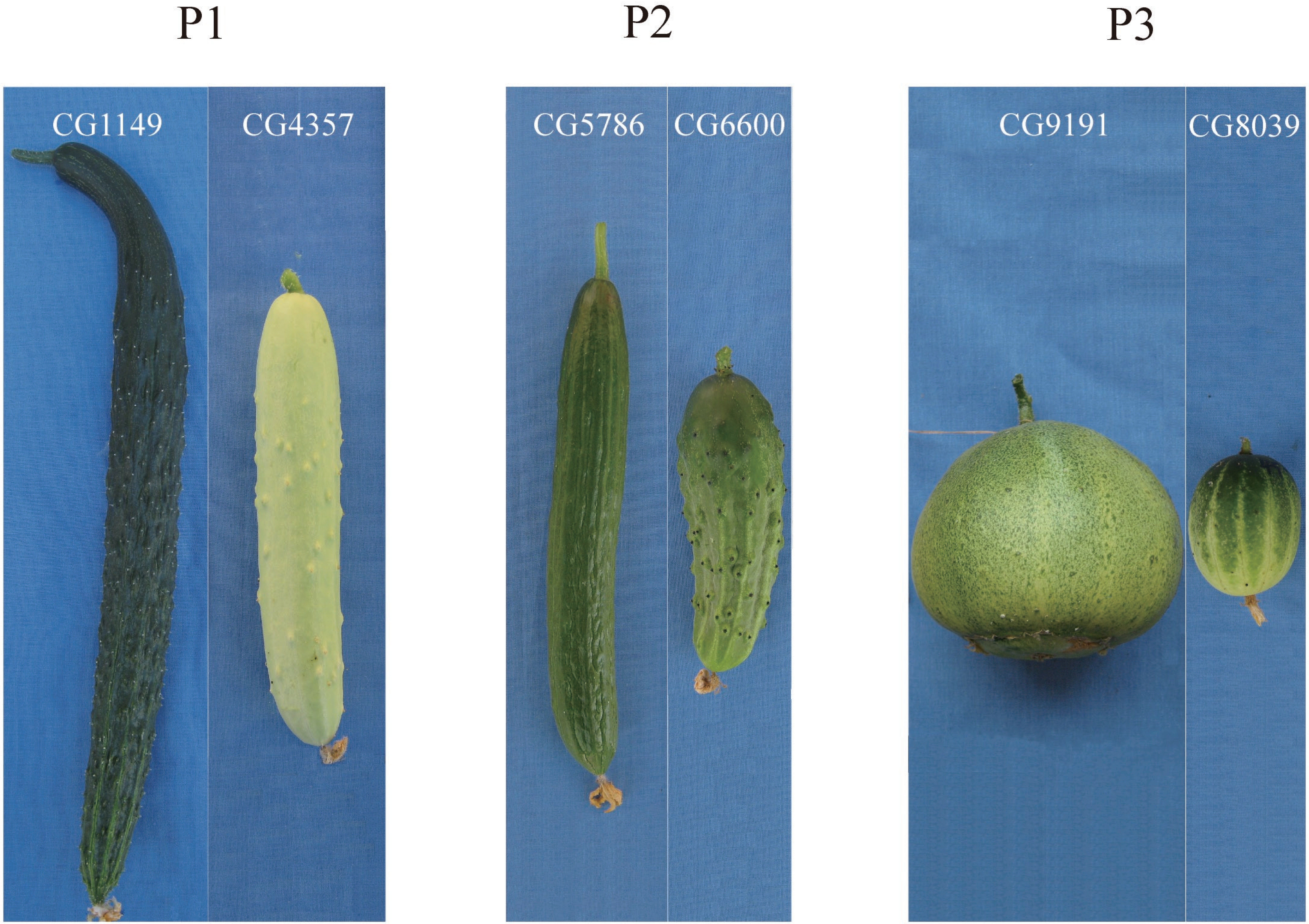

Legend: CG1149, "Chinese Long"; CG4357, Southern China type; CG5786, Dutch greenhouse; CG6600, Pickling; CG9191, Xishuangbanna; CG8039, Indian
